# Circular RNA profiling identifies circ_0001522, circ_0001278, and circ_0001801 as predictors of unfavorable prognosis and drivers of triple-negative breast cancer hallmarks

**DOI:** 10.1038/s41420-025-02576-9

**Published:** 2025-07-09

**Authors:** Dania Awata, Vishnubalaji Radhakrishnan, Hibah Shaath, Ramesh Elango, Sameera Rashid, Mohammed Akhtar, Thasni Karedath Abdul Azis, Ikhlak Ahmed, Khalid Ouararhni, Ammira S. Al-Shabeeb Akil, Nehad M. Alajez

**Affiliations:** 1 College of Health & Life Sciences, Hamad Bin Khalifa University (HBKU), Qatar Foundation (QF), Doha, Qatar; 2 Translational Oncology Research Center (TORC), Qatar Biomedical Research Institute (QBRI), Hamad Bin Khalifa University (HBKU), Qatar Foundation (QF), Doha, Qatar; 3 Royal Liverpool university NHS foundation trust, Liverpool, UK; 4 Department of Laboratory Medicine, Hamad Medical Corporation (HMC), Doha, Qatar; 5 Genomics Core Facility, Hamad Bin Khalifa University, Qatar Foundation, Doha, Qatar; 6 Precision OMICs Research & Translational Science Lab, Sidra Medicine, Doha, Qatar; 7 Metabolic and Mendelian Disorders Clinical Research Program, Sidra Medicine, Doha, Qatar

**Keywords:** Breast cancer, Small RNAs

## Abstract

Breast cancer poses a significant clinical challenge due to its complex molecular landscape, underscoring the need for improved prognostic and therapeutic strategies. In this study, we explored the expression profiles and therapeutic relevance of circular RNAs (circRNAs) in a cohort of 96 breast cancer patients from Qatar representing the MENA region. Our data identified distinct expression patterns in relation to breast cancer subtypes, tumor grade, and age, with fifty circRNAs found to be associated with unfavorable relapse-free survival (RFS). The expression of sixteen of these circRNAs was validated in triple-negative breast cancer (TNBC) model using RNase R resistance assay. Among these, the expression of circ_0001522, circ_0001278, and circ_0001801 was validated using divergent primers, and their backsplice junctions were confirmed using Sanger sequencing. Functionally, siRNA-mediated knockdown of these circRNAs significantly suppressed cell proliferation, colony formation, three-dimensional organoid growth, and cell migration in TNBC models. Mechanistic investigations revealed that circRNA depletion altered a subset of miRNA and mRNA expressions, with key interactions involving miR-4458, miR-145-5p, and miR-760, regulating critical targets such as CCND1, ROBO4, and MMP1. Additionally, circRNA-RBP bioinformatic analysis identified common binding partners, including AGO2, CPSF7, TARDBP, UPF1, and LIN28B, suggesting roles in post-transcriptional regulation. Our data highlight circ_0001522, circ_0001278, and circ_0001801 as promising prognostic and therapeutic circRNA targets for breast cancer, offering new avenues for improving breast cancer prognosis and treatment.

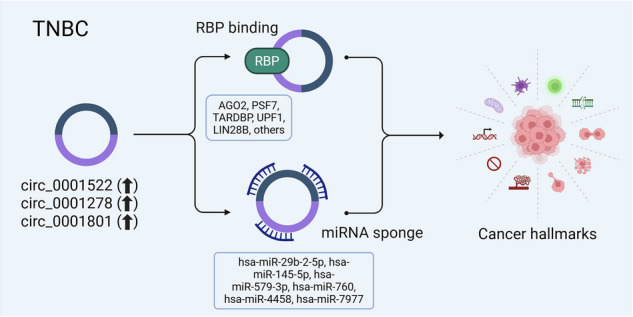

## Introduction

Breast cancer remains the most commonly diagnosed malignancy and the leading cause of cancer-related deaths among women globally [1–3]. Despite advances in diagnostic and therapeutic strategies, the lack of robust biomarkers and effective treatments, especially for the aggressive triple-negative breast cancer (TNBC) subtype, continues to be a significant challenge. Currently there is limited knowledge about the biology of breast cancer from the Middle East and North Africa (MENA) region. We recently performed transcriptomic profiling and identified the expression patterns of mRNAs, microRNAs (miRNAs), and long noncoding RNAs in breast cancer samples from the MENA region [4, 5].

Non-coding RNAs, including circular RNAs (circRNAs), have emerged as key regulatory elements in the context of breast cancer progression. CircRNAs are unique due to their covalently closed loop structures, which confer stability and resistance to degradation, distinguishing them from linear RNAs. These molecules are increasingly recognized for their roles in cancer biology, where they modulate gene expression, cancer progression, and drug resistance [6–10]. These circRNAs predominantly act as miRNA sponges, modulating miRNA-mediated gene regulation and affecting cancer-related pathways. Additionally, circRNAs can interact with specific RNA-binding proteins (RBPs), influencing key signaling pathways involved in breast cancer [10]. CircRNAs have also been implicated in breast cancer drug resistance, particularly chemotherapy resistance, by modulating cell death mechanisms, cell proliferation, and the expression of drug resistance genes [8, 11, 12]. Due to their stability, abundance, and tissue-specific expression, circRNAs are considered promising biomarkers for cancer diagnosis, prognosis, and therapeutic targeting. For instance, Liu et al. reported circ_001783 to promote breast cancer progression by sponging miR-200c-3p, correlating with greater tumor burden and poorer prognosis. Circ_001783 knockdown inhibited cell proliferation and invasion, making it a potential prognostic and therapeutic target [13]. Du et al. reported low circ-Foxo3 expression in breast cancer cells, which increased during apoptosis. Circ-Foxo3 enhances Foxo3 protein levels while promoting MDM2-mediated p53 degradation, leading to apoptosis via PUMA upregulation. Its overexpression inhibits tumor growth, highlighting its potential regulatory role in cancer [14]. Wang et al. conducted a comprehensive analysis of circRNA expression across seven solid tumors, identifying both cancer-specific and common dysregulated circRNAs. The authors validated circLIFR (circ_0072309) as downregulated in multiple cancers, showing inhibition of cell migration and metastasis, highlighting circRNAs’ broad involvement in tumor progression [15]. While those studies provide insight on the role of various circRNA in breast cancer, the expression patterns of circRNAs in breast cancer from underrepresented regions, including the MENA, remains unexplored. Additionally, the functionality of the vast majority of circRNAs remains largely unknown.

Herin, we conducted circRNA profiling on 96 breast cancer samples from Qatar representing the MENA region and assessed their prognostic association with relapse-free survival (RFS). Selected circRNAs were validated for their expression and resistance to RNase R treatment in TNBC models using RNA-Seq and divergent primers PCR. Interestingly, targeted depletion of a subset of RFS-associated circRNAs (circ_0001522, circ_0001278, and circ_0001801) led to significant reductions in TNBC colony formation and growth suppression under three-dimensional (3D) culture conditions. Mechanistically, transcriptomic and bioinformatics analysis suggested interactions between these circRNAs and key miRNAs (miR-4458, miR-145-5p, miR-760) and downstream targets (CCND1, ROBO4, MMP1), along with potential binding to several RBPs including AGO2, LIN28B, and IGF2BP2, among others, highlighting their roles in post-transcriptional gene regulation.

Our data established a comprehensive map of circRNA expression in breast cancer and set a foundation for developing novel prognostic biomarkers and therapeutic strategies for breast cancer patients.

## Results

### circRNA expression profiling and prognostic analysis in breast cancer

**Fig. 1:**
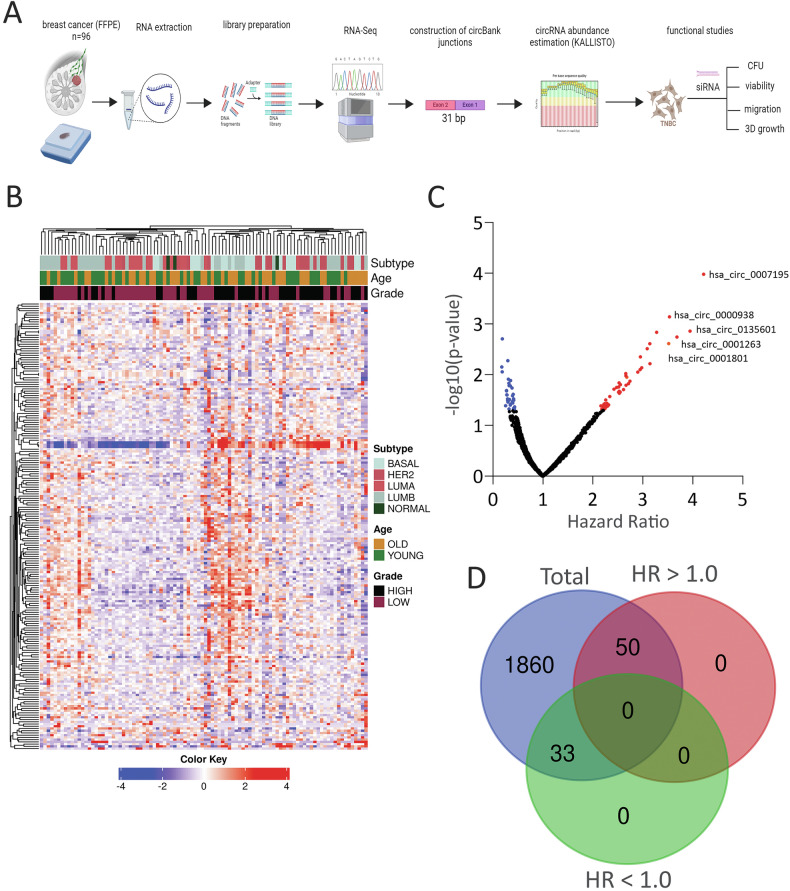
CircRNA profiling of breast cancer and survival analysis. **A** Schema illustrating the overall study design. **B** Heatmap showing clustering of breast cancer (*n* = 96) in relation to breast cancer subtypes, tumor grade, and age based on circRNA expression. **C** Volcano plot displaying circRNAs associated with worse (red) or better (blue) prognosis based on relapse-free survival (RFS) analysis. X-axis represents hazard ratio (HR), and y-axis represents -log10 p-value. Top five circRNAs correlating with worse prognosis are labeled. **D** Venn diagram showing the number of circRNAs linked to worse (HR > 1.0) and better (HR < 1.0) prognosis (*p* < 0.05) among total expressed circRNAs in the cohort.

The experimental approach for circRNA expression profiling and functional analysis is illustrated in Fig. 1A. To provide comprehensive map of circRNA expressions in breast cancer from our cohort, we performed circRNA expression profiling on 96 breast cancer samples, assessing the abundance of 22,076 circRNA transcripts annotated in the circBank database. A heatmap depicting the changes in circRNA expression in relation to breast cancer subtypes, tumor grade, and age is shown in Fig. 1B. To identify circRNA transcripts associated with breast cancer prognosis, we performed Kaplan-Meier survival analysis on log2-transformed expression data covering 1943 expressed circRNAs from our cohort. This analysis identified 50 circRNAs associated with poor prognosis (HR > 1.0) and 33 circRNAs predicting better prognosis (HR < 1.0), as illustrated in Fig. 1C, D.

### Validation of circRNA candidates using RNase R resistance assay

**Fig. 2:**
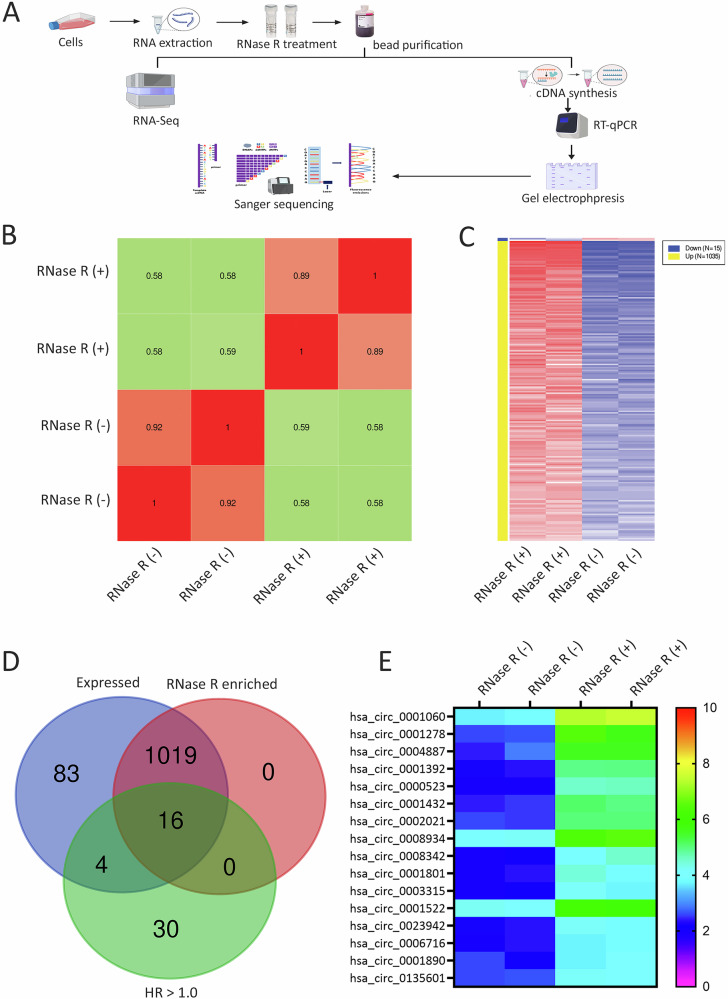
Identification of circRNAs expressed by TNBC models using RNase R assay. **A** Schema illustrating the RNase R assay strategy and NGS workflow. **B** Correlation matrix showing correlation scores for RNase R-treated (*n* = 2) and control (*n* = 2) MDA-MB-231 samples from RNA-Seq analysis. **C** Heatmap displaying differentially expressed circRNAs in RNase R-treated versus control MDA-MB-231 samples. **D** Venn diagram showing the intersection between RNase R-enriched circRNAs and circRNAs associated with worse prognosis (HR > 1.0), relative to the total circRNAs expressed in MDA-MB-231 cells. **E** Heatmap showing expression of 16 circRNAs linked to worse prognosis and enriched by RNase R treatment.

RNase R resistance assay has previously been shown to enrich circRNAs, while linear RNAs are sensitive to this treatment [16]. The experimental approach to validate circRNA resistance to RNase R treatment is illustrated in Fig. 2A. To enrich the circRNA fraction in RNA samples from TNBC model, total RNA was extracted from MDA-MB-231 cells and then treated with RNase R, followed by SPRI bead purification. The extracted RNA was subjected to total RNA-Seq and the circRNA abundance was subsequently compared to RNA-Seq data from untreated RNA extracted from MDA-MB-231 cells. FASTQ data generated from MDA-MB-231 cells (RNase R treated (+) and RNase R untreated (-)) were aligned to the Kallisto circBank index. CircRNA expression was normalized to the linear mRNA transcriptome from GENCODE release 45. Correlation analysis revealed strong concordance between technical replicates (Fig. 2B), where least correlation was observed in RNase R+ compared to RNase R- samples. Differentially expressed circRNAs in RNase R+ samples compared to RNase R- samples are presented as a heatmap (Fig. 2C). The overlap between expressed circRNAs, RNase R-enriched circRNAs, and circRNAs correlating with poor prognosis (HR > 1.0) is shown in the venn diagram in Fig. 2D. This analysis identified 16 enriched circRNAs associated with worse prognosis in breast cancer. The relative expression of those 16 circRNAs in RNase R+ vs RNase R- samples is depicted in the heatmap in Fig. 2E.

### Validation of circRNA junctions using Sanger sequencing

**Fig. 3:**
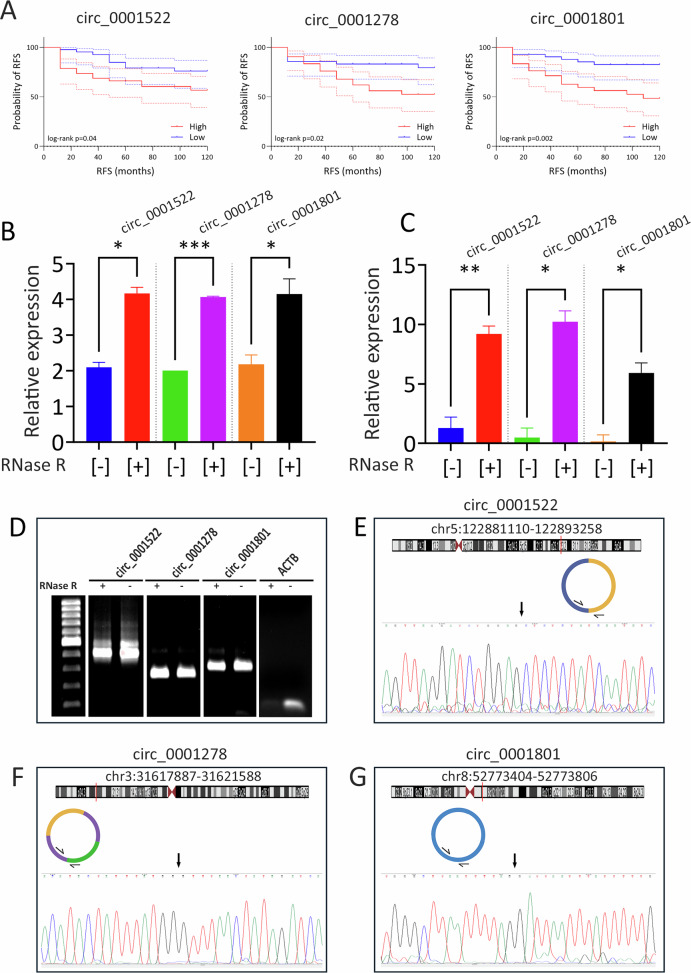
Survival analysis and validation of backsplice junctions of shortlisted circRNA candidates using Sanger sequencing. **A** Ten-year Kaplan–Meier RFS curves for circ_0001522, circ_0001278, and circ_0001801. Patients were stratified into high vs low based on median circRNA expression. **B** Relative expression of circ_0001522, circ_0001278, and circ_0001801 from RNA-Seq analysis. Data are shown as mean ± SD, *n* = 2. Expression levels were normalized to the GENCODE release 45 linear transcriptome. **C** Validation of circRNA expression using divergent primers, normalized to ACTB expression. Data are shown as mean ± SEM, *n* = 6. **D** Gel electrophoresis showing correct PCR amplicon sizes for each circRNA with (+) and without (-) RNase R treatment. Sanger sequencing confirms backsplice junctions for circ_0001522 (**E**), circ_0001278 (**F**), and circ_0001801 (**G**). *p < 0.05, **p < 0.005, ***p < 0.0005.

Among the identified circRNAs, three (circ_0001522, circ_0001278, and circ_0001801) were selected for further investigation due to their novel association with breast cancer. The 10-year Kaplan-Meier RFS curves for circ_0001522, circ_0001278, and circ_0001801 are shown in Fig. 3A, indicating poor RFS in breast cancer patients overexpressing these three circRNAs. As shown in Fig. 3B, elevated expression of these circRNAs was observed in RNase R+ samples based on RNA-Seq data. To validate their enriched expression in RNase R+ samples, RT-qPCR was performed on those samples from TNBC models using divergent primers, with ACTB serving as a reference gene. Data presented in Fig. 3C confirmed significant enrichment of the three circRNAs in RNase R+ samples, thus validating the RNA-Seq data. Semi-quantitative PCR and gel electrophoresis confirmed the expected band size for each circRNA junction detected in both RNase R+ and RNase R- samples, whereas the ACTB linear amplicons were more abundant in RNase R- samples (Fig. 3D). Sanger sequencing further validated the backsplice junction for each of the three circRNAs (Fig. 3E–G). Taken together, our data confirmed the presence and integrity of the backsplice junctions for circ_0001522, circ_0001278, and circ_0001801 in TNBC models.

### Targeted depletion of circRNAs impair TNBC proliferation

**Fig. 4:**
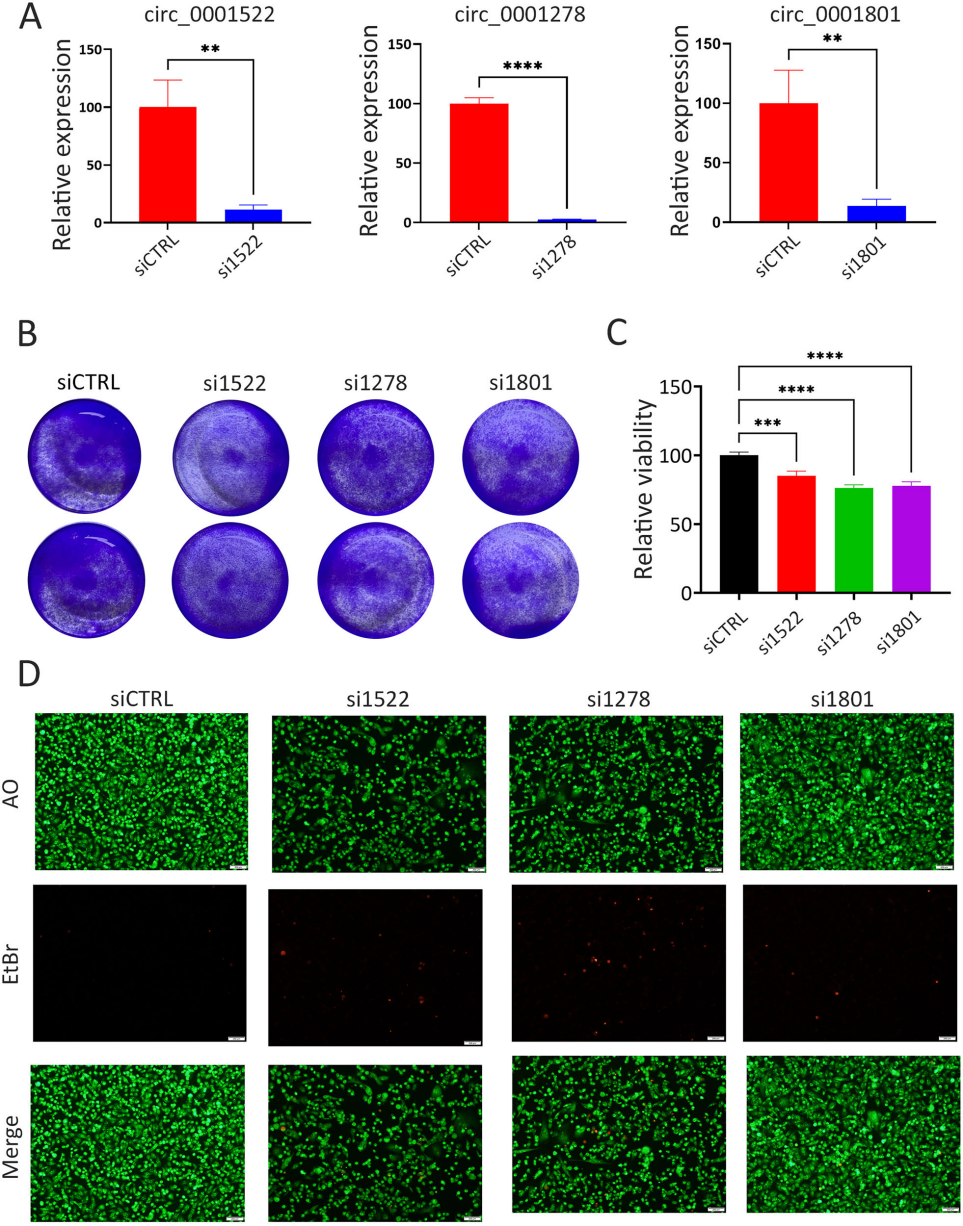
Functional consequences of circRNA knockdown on TNBC proliferation and viability. **A** Efficient knockdown of circ_0001522, circ_0001278, and circ_0001801 using siRNAs targeting the respective backsplice junctions. Data are shown as mean ± SEM, *n* = 9. **B** Representative images from colony-forming unit (CFU) assay demonstrating suppression of CFU potential in MDA-MB-231 cells by siRNA-mediated silencing of circ_0001522, circ_0001278, and circ_0001801. Two technical replicas images are shown for each condition. **C** Quantification of MDA-MB-231 CFU potential following depletion of the indicated circRNAs. Data are shown as mean ± SEM, *n* = 18 from three independent experiments. **D** Representative images showing AO (upper panel), EtBr (middle panel), and merged (lower panel) staining in MDA-MB-231 cells transfected with siControl (siCTRL), siCirc_0001522 (si1522), siCirc_0001278 (si1278), and siCirc_0001801 (si1801). **p < 0.005, ***p < 0.0005, ****p < 0.00005.

To elucidate the functional significance of the identified circRNAs in TNBC, siRNA-mediated silencing was used to knock down the expression of the three selected circRNAs by targeting their corresponding backspliced junctions in the MDA-MB-231 and BT-549 TNBC models. As shown in Fig. 4A, the indicated siRNAs effectively suppressed the expression of the targeted circRNAs. Similar results were also observed in the BT-549 model (Fig. S1A). This targeted depletion of circ_0001522, circ_0001278, and circ_0001801 led to a marked inhibition of CFU potential in both MDA-MB-231 (Fig. 4B) and BT-549 (Fig. S1B) TNBC models. Quantitative analysis further demonstrated a significant reduction in CFU potential in MDA-MB-231 (Fig. 4C) and BT-549 (Fig. S1C) cells following the knockdown of these circRNAs. Live and dead imaging further confirmed the suppression of cell proliferation and the induction of cell death in MDA-MB-231 cells transfected with siRNAs targeting circ_0001522, circ_0001278, and circ_0001801, compared to cells treated with siCTRL (Fig. 4D). Similar results were also observed in the BT-549 model (Fig. S2). These findings underscore the crucial role of the targeted circRNAs in sustaining TNBC cell viability and highlight their potential as therapeutic targets in disrupting TNBC proliferation and tumorigenic capacity.

### Suppression of three-dimensional growth and cell migration in TNBC cells depleted of circ_0001522, circ_0001278, and circ_0001801

**Fig. 5:**
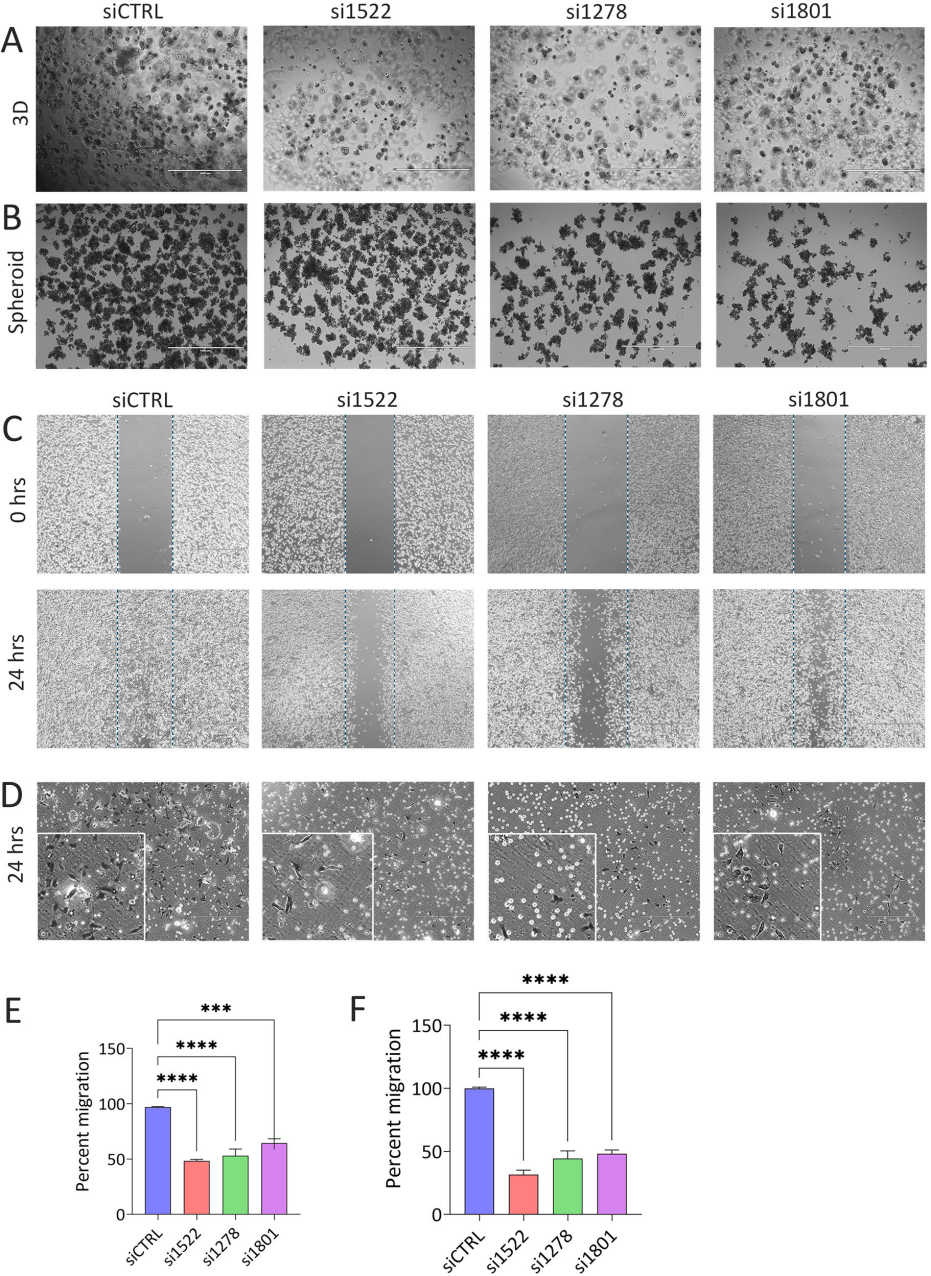
Targeted depletion of circ_0001522, circ_0001278, and circ_0001801 inhibits 3D growth and cell migration in TNBC. **A** Representative images showing suppression of MDA-MB-231 cell growth in 3D Matrigel cultures following depletion of the indicated circRNAs. **B** Representative images showing spheroid formation in MDA-MB-231 cells after depletion of circ_0001522, circ_0001278, and circ_0001801 compared to siCTRL cells. Migration assays showing reduced migration potential of TNBC cells upon circRNA depletion, assessed by wound healing (**C**) and Boyden chamber (**D**) assays. Quantification of migration from wound healing (**E**) and Boyden chamber (**F**) assays. Data are shown as mean ± SEM, *n* = 3. ***p < 0.0005, ****p < 0.00005.

In agreement with the CFU data demonstrating the inhibition of TNBC growth under 2D settings, depletion of these circRNAs in MDA-MB-231 and BT-549 cells significantly reduced 3D growth in Matrigel, as shown in Fig. 5A and Fig. S3A, respectively. Similarly, spheroid formation was markedly suppressed in MDA-MB-231 and BT-549 cells following circRNA knockdown compared to siCTRL cells (Figs. 5B and S3B). We next assessed the impact of circRNA depletion on cell migration. Wound healing assays demonstrated reduced migration capacity in MDA-MB-231 TNBC cells upon depletion of circ_0001522, circ_0001278, and circ_0001801 (Fig. 5C). This reduction was further confirmed using Boyden chamber migration assays (Fig. 5D). Quantification of cell migration from both assays showed a significant decrease in migratory potential in circRNA depleted MDA-MB-231 TNBC cells (Fig. 5E, F).

### Identification of circRNA-miRNA-mRNA regulatory networks

**Fig. 6:**
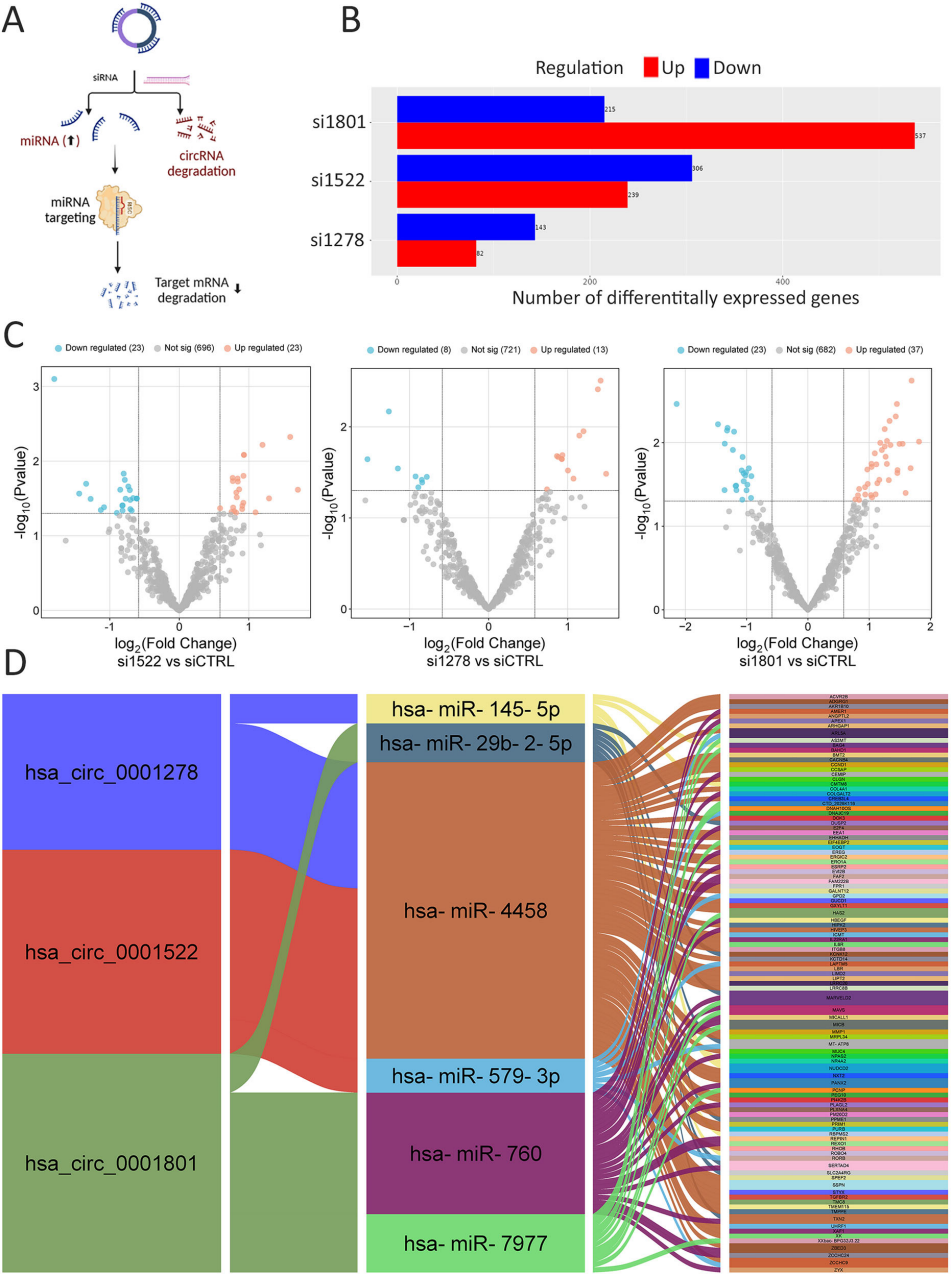
Identification of circRNA-miRNA-mRNA regulatory networks. **A** Schema depicting the approach used to identify circRNA-miRNA-mRNA regulatory networks for the three circRNA candidates. **B** Bar chart showing the number of upregulated (red) and downregulated (blue) mRNAs following depletion of circ_0001522, circ_0001278, and circ_0001801 compared to siCTRL cells. **C** Volcano plots showing upregulated and downregulated miRNAs in cells depleted of the indicated circRNAs. **D** Alluvial plot depicting interactions between circ_0001522, circ_0001278, and circ_0001801 and miRNAs (miR-4458, miR-145-5p, miR-760, miR-7977, miR-579-3p) and their gene targets (ADGRG1, RHOB, UHRF1, ROBO4, CCND1, GXYLT1, EOGT, TMPPE, FAM222B, MICALL1, NPAS2, SERTAD4, TXN2, ARHGAP1, EIF4EBP2, MAVS, E2F4, HBEGF, PRIM1, TGFBR2, COL4A1, EHHADH, ERO1A, ICMT, LIMD2, LIPT2, TMC8, ZCCHC9, RORB, and LBR).

A schematic overview of the approach used to construct circRNA-miRNA-mRNA interaction networks is shown in Fig. 6A. To elucidate the mechanisms by which these circRNAs contribute to TNBC pathogenesis, MDA-MB-231 cells were transfected with siRNAs targeting circ_0001522, circ_0001278, and circ_0001801 along with control siRNA. Following transfection, total RNA and microRNA profiling were performed using NGS. Our analysis identified 306, 143, and 215 downregulated mRNAs in MDA-MB-231 cells depleted of circ_0001522, circ_0001278, and circ_0001801, respectively (Fig. 6B). Additionally, 23, 13, and 37 miRNAs were upregulated in cells transfected with siRNAs targeting circ_0001522, circ_0001278, and circ_0001801, respectively (Fig. 6C). Using Miranda 3.3a and RNA22v2 algorithms, we conducted miRNA-circRNA prediction analysis to identify miRNAs predicted to directly interact with these circRNAs. The analysis revealed circ_0001522 to interact with miR-579-3p and miR-4458, while circ_0001278 was found to interact with let-7c-5p, miR-145-5p, and miR-4458. Additionally, circ_0001801 was found to interact with miR-29b-2-5p, miR-760, and miR-7977. The identified miRNAs were further analyzed using the miRNA target filter tool in IPA, enabling the elucidation of the miRNA-mRNA regulatory networks associated with each circRNA. An Alluvial plot illustrating the circRNA-miRNA-mRNA interactions derived from our analysis is presented in Fig. 6D, collectively suggesting that these circRNAs function as miRNA sponges to regulate gene expression in TNBC. Validation of downregulated expression of MMP1 and CCND1 in circRNA knockdown cells is shown in Fig. S4.

### Identification of circRNA-RBP regulatory networks

**Fig. 7:**
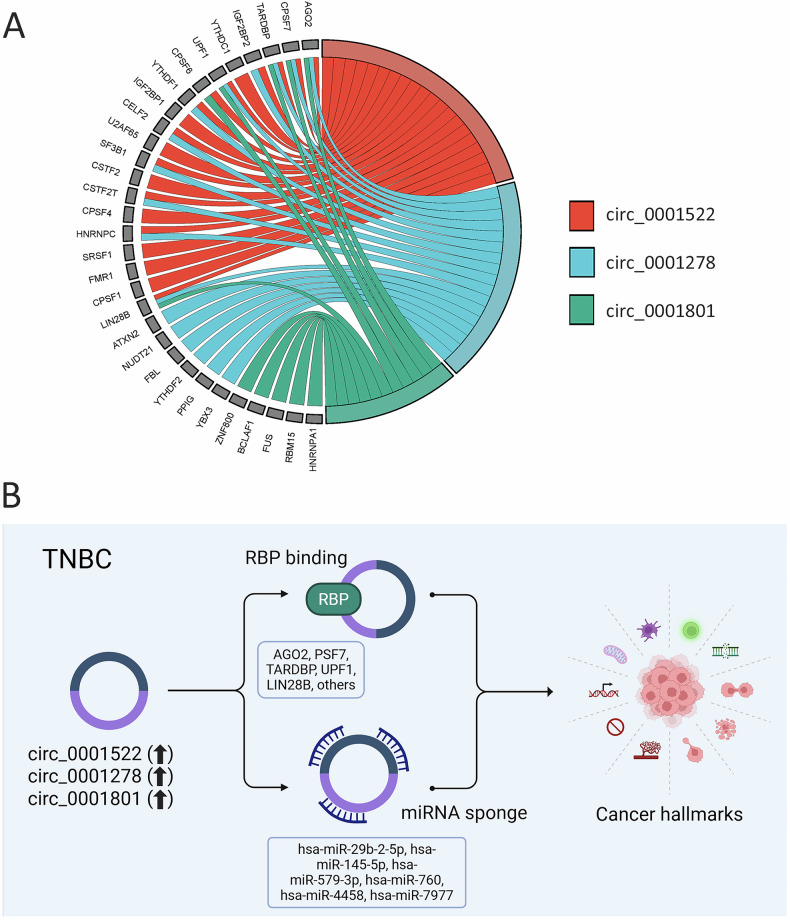
Identification of circRNA-RBP regulatory networks in TNBC. **A** Circos plot illustrating interactions between circ_0001522, circ_0001278, and circ_0001801 and RNA-binding proteins (RBPs) including AGO2, CPSF7, TARDBP, IGF2BP2, YTHDC1, UPF1, CPSF6, YTHDF1, IGF2BP1, CELF2, U2AF65, SF3B1, CSTF2, CSTF2T, CPSF4, HNRNPC, SRSF1, FMR1, CPSF1, LIN28B, ATXN2, NUDT21, FBL, YTHDF2, PPIG, YBX3, ZNF800, BCLAF1, FUS, RBM15, and HNRNPA1. **B** Schema depicting the role of circ_0001522, circ_0001278, and circ_0001801 in regulating TNBC hallmarks via miRNA sponging and RBP interactions.

CircRNAs can also exert their functions through interactions with RBPs. Analysis of the CircAtlas 3.0 database revealed that circ_0001522 interacts with 20 RBPs, circ_0001278 interacts with 17, and circ_0001801 was found to interact with 11 RBPs (Fig. 7A and Table S3). Notably, all three circRNAs were predicted to interact with a shared set of RBPs (AGO2, CPSF7, TARDBP, UPF1, and LIN28B) as illustrated in the circos plot (Fig. 7A). Figure 7B presents a model summarizing the role of those three onco-circRNAs in modulating TNBC hallmarks through miRNA sponging and RBP interactions. These findings highlight the complex regulatory networks mediated by these circRNAs, contributing to TNBC progression through their roles in cell growth, migration, and gene regulation.

## Discussion

Breast cancer remains one of the most prevalent and aggressive cancers affecting women worldwide, with a high mortality rate underscoring the need for novel targeted therapeutic strategies [2, 17]. Among the many types of ncRNAs, circRNAs have emerged as key players in various cancers, including breast cancer [18]. The identification of circRNAs associated with breast cancer prognosis represents a critical advancement in understanding tumor biology, offering promising avenues for the development of novel prognostic biomarkers and targeted therapeutic strategies. In this study, we combined circRNA profiling from 96 patient samples from Qatar, representing the wider MEAN region, with computational and RNase R enrichment analyses to map circRNA expression landscapes and assess their prognostic significance. Our analysis of 22,076 circRNA transcripts identified numerous circRNAs where their expression correlated with breast cancer subtypes, tumor grade and age, consistent with prior studies reporting widespread circRNA dysregulation in cancer [19].

CircRNAs exhibit resistance to exonuclease digestion due to their closed-loop structure, rendering them more stable than linear RNAs [20]. Consistent with this property, our results confirmed that hundreds of circRNAs were significantly enriched in RNase R-treated samples. Survival analysis revealed 50 circRNAs to be associated with poor prognosis, of which 16 circRNAs, including circ_0001522, circ_0001278, and circ_0001801, were detected in TNBC models and were validated as RNase R-resistant. RT-PCR with divergent primers relative to ACTB and Sanger sequencing confirmed the enrichment and presence of back-splice junctions for these circRNAs.

To investigate their functional roles in TNBC, we performed siRNA-mediated knockdown of circ_0001522, circ_0001278, and circ_0001801 in MDA-MB-231 and BT-549 cells. Knockdown of the three circRNAs significantly inhibited colony formation, suppressed 3D growth in Matrigel, and reduced spheroid formation. Moreover, wound healing and Boyden chamber assays demonstrated markedly impaired cell migration following circRNA depletion. These findings highlight the critical role of these circRNAs in promoting TNBC cell proliferation and migration, key processes in cancer progression.

Our study further explored the molecular mechanisms underlying the oncogenic roles of these circRNAs. Comprehensive RNA and microRNA profiling identified substantial downregulation of mRNAs and upregulation of miRNAs in circRNA-depleted TNBC cells. Notably, miRNA prediction analysis revealed that circ_0001801 sponges miR-29b-2-5p, miR-760, and miR-7977; circ_0001278 sponges let-7c-5p, miR-145-5p, and miR-4458; while circ_0001522 sponges miR-579-3p and miR-4458. These interactions suggest that circRNAs act as miRNA sponges, regulating gene expression in TNBC. Network analyses further delineated the circRNA-miRNA-mRNA interactions, implicating these circRNAs in modulating key pathways associated with TNBC pathogenesis.

CircRNAs also interact with RBPs to influence cellular processes. Using CircAtlas 3.0 database, we identified multiple RBP interactions for circ_0001522, circ_0001278, and circ_0001801, including AGO2, CPSF7, TARDBP, UPF1, and LIN28B, supporting a dual regulatory mechanism involving miRNA sponging and RBP binding. This duality may underlie their robust effects on TNBC growth and migration.

These findings align with previous reports highlighting the oncogenic potential of these circRNAs in other cancers. For example, Li et al. demonstrated that circ_0001522 (circCSNK1G3) promotes tumor proliferation and metastasis in renal cell carcinoma via miR-181b-mediated EMT regulation [21]. Chen et al. similarly linked circCSNK1G3 to prostate cancer progression [22]. Ge et al. showed that circ_0001278 regulates colorectal cancer progression by sponging miR-338-5p [23], while Zheng et al. reported that circ_0001801 (circPCMTD1) enhances glioma growth by interacting with miR-224-5p [24]. Our study provides additional evidence supporting the oncogenic roles of these circRNAs in TNBC. While our findings demonstrate the functional relevance of circ_0001522, circ_0001278, and circ_0001801 in TNBC cancer hallmarks through in vitro assays, further investigations using in vivo models are warranted to validate their prognostic and therapeutic potential within a more physiologically relevant context.

## Conclusions

Collectively, our results highlight the prognostic and functional importance of circ_0001522, circ_0001278, and circ_0001801 in TNBC proliferation, migration, and gene regulation. The significant upregulation and regulatory potential of these circRNAs underscore their prognostic relevance and potential as therapeutic targets. Future research should further explore the therapeutic utility of targeting circRNA-mediated networks in TNBC.

## Methods

### Study cohort and ethical declaration

This study was conducted on 96 FFPE samples from treatment-naïve breast cancer patients from Hamad Medical Corporation (HMC). Details regarding the exclusion and inclusion criteria for the study cohort are provided in our recent publication [4]. Briefly, eligible cases included female patients with invasive breast carcinoma who underwent primary tumor excision at HMC between 2008 and 2013. Cases were excluded if there was prior neoadjuvant chemotherapy, in-situ carcinoma, male gender, recurrent breast cancer, sarcoma, or metastasis from a non-breast primary tumor. The study was approved by the ethical committees of Hamad Medical Corporation, Doha, Qatar (MRC-01–19–142) and Qatar Biomedical Research Institute, Doha, Qatar (QBRI-IRB 2020–09–035). All experiments were conducted in accordance with the ethical guidelines outlined in the Declaration of Helsinki.

### Sample preparation

Total RNA was extracted from FFPE core punches using the recover all total nucleic acid isolation kit (Ambion Inc., Life Technologies, USA) as we described before [4]. Briefly, core tissue samples weighing less than 35 mg were placed in a mortar with liquid nitrogen and finely ground using a pestle. The samples were then deparaffinized by incubating in xylene for 3 min at 50 °C, followed by centrifugation and two washes with 100% ethanol. The pellets were vacuum-dried, and proteins were then degraded by incubation with protease for 15 min at 50 °C and then for another 15 min at 80 °C. Nucleic acids were captured using columns and were treated with DNAse, and the RNA was subsequently eluted in nuclease-free water. The concentration and purity of the extracted RNA were measured using a NanoDrop 2000 (Thermo Scientific, DE, USA), and the RNA was stored at −80 °C until further use.

### Library preparation and sequencing

Total RNA was used to prepare libraries employing the TruSeq Stranded Total RNA Library Kit (Cat no. 20020597, Illumina Inc., San Diego, CA, USA) according to the manufacturer’s protocol. Briefly, 500 ng of total RNA underwent rRNA depletion and First-strand cDNA synthesis was performed using random hexamers and SuperScript II Reverse Transcriptase (Cat#: 18064014, Thermo Fisher Scientific, Waltham, MA, USA). Second-strand cDNA synthesis was carried out with dUTP replacing dTTP. Double-stranded cDNA was end-repaired, adenylated, and ligated to barcoded DNA adapters before amplification. The library quality was checked using an Agilent 2100 Bioanalyzer system and quantified with a Qubit system. The libraries were pooled, clustered on a cBot platform, and sequenced on an Illumina HiSeq 4000, generating approximately 50 million paired-end reads (2 × 75 bp) per sample. The RNA-seq data generated in this study have been deposited in the SRA repository under BioProject number PRJNA954402.

### Library preparation from RNase R-treated samples

For library preparation from RNase R treatment, 2 micrograms of total RNA were incubated with 2 μL of RNase R buffer (ab286929, Abcam), 0.5 μL of RNaseOUT™ (40U/μL) Ribonuclease Inhibitor (Invitrogen), and 2.5 μL (10 U/μL) of RNase R enzyme (ab286929, Abcam) at 37 °C for 30 minutes. Purification was performed using SPRI beads (2.5×, Beckman Coulter) according to the manufacturer’s protocol. Subsequently, 2 micrograms of total RNA from RNase R-treated and non-treated MDA-MB-231 cells were used for library preparation. First-strand cDNA synthesis was performed using random hexamers and SuperScript II Reverse Transcriptase. Second-strand cDNA was synthesized with dUTP replacing dTTP, followed by end-repair, adenylation, and ligation of barcoded DNA adapters to both ends of the double-stranded cDNA. The library quality was assessed using the Agilent 2100 Bioanalyzer system, and quantification was performed using the Qubit 2.0 fluorometer (Invitrogen). RNA sequencing was performed on an Illumina HiSeq instrument as described above.

### CircRNA quantification using Kallisto

A reference backsplice junction was generated using annotated circRNA FASTA sequences from the circBank database [25]. Each backsplice junction was created by ligating the last 15 bp of the transcript’s end with the first 16 bp from the beginning, forming the circular junction reference, using Biopython library (Python 3.11). A Kallisto index was then constructed using the circRNA putative junctions and a k-mer length of 31, as previously described [26, 27]. RNA-seq data (FASTQ) from the 96 tumor samples were subsequently aligned to Kallisto index file and abundance estimation of each circRNA junction was computed.

### CircRNA differential expression analysis

Differential expression of circRNAs was conducted using iDEP 2.0 as described previously [4]. Expression data (total counts) were imported into iDEP 2.0 and were first normalized (CPM, count per million) and then data transformation was conducted using EdgeR (log2(CPM+c)). CircRNA expression in relation to breast cancer subtype, tumor grade, and age were assessed using iDEP 2.0.

### Validation of circRNA candidates using RNase R assay

To validate expression of circRNA candidates in TNBC models, the exoribonuclease enzyme RNase R was used to digest total RNA from MDA-MB-231 TNBC model, followed by high-throughput NGS sequencing for both RNase R-treated and untreated samples. The Kallisto algorithm was applied to detect circRNA enrichment by comparing the RNase R-treated samples to the non-treated control samples, mapped against the 31 bp junction index. Divergent primers and RT-qPCR were used to detect circular junctions, relative to linear housekeeping gene (ACTB) using the 2−ΔΔct method. The sequences of primers used in this study are listed in Table S1.

### Survival analysis

To identify circRNAs significantly associated with ten-year patients’ RFS, normalized circRNA expression data (log2) were subjected to survival analysis using the ‘survival’ package in RStudio 2023.12.1. Survival curves for breast cancer samples were generated using the Kaplan-Meier function and differences were analyzed using the log-rank test in IBM SPSS statistics software (SPSS, Chicago, Illinois, USA). A log-rank *p*-value of < 0.05 was considered statistically significant, while accounting for potential confounding factors such as age, tumor grade, and molecular subtype. Graphing was performed using GraphPad Prism v9.

### Sanger sequencing

The circRNA junctions for circ_0001522, circ_0001278, and circ_0001801 were amplified using specific divergent primers and platinum Taq DNA Polymerase High Fidelity (Invitrogen). The sizes of the PCR amplicons were confirmed by gel electrophoresis, followed by gel extraction and Sanger sequencing on an Applied Biosystems 3130xl Genetic Analyzer. The resulting data were analyzed using the ChromasPro software (Technelysium Pty Ltd, v1.5) to validate the sequence and visualize the junctions.

### Identification of potential circRNA-binding miRNAs and their gene targets

The binding interactions between upregulated miRNAs in circRNA knockdown cells and circRNAs were assessed using RNA22v2 and miRanda 3.3a, following previously described methods [28–30]. Only candidates predicted by both algorithms were included in the final analysis. Next, we identified mRNAs that were downregulated in circRNA knockdown cells. The lists of upregulated miRNAs (also predicted by RNA22 and miRanda) and the corresponding downregulated mRNAs were then imported into Ingenuity Pathway Analysis (IPA) software (Ingenuity Systems; Qiagen, Redwood City, CA, USA). Using the microRNA Target Filter tool in IPA, we identified the respective mRNA targets (experimentally observed and predicted (high confidence)) and subsequently constructed circRNA-miRNA-mRNA networks as we described before [4].

### Construction of circRNA-RBP networks

To construct the circRNA-RBP network, the lists of RBPs interacting with circ_0001522, circ_0001278, and circ_0001801 were retrieved from the CircAtlas 3.0 database [31]. Subsequently, a chord plot illustrating the interactions between the RBPs and their respective circRNAs was generated using SRplot, as previously described [30].

### Cell culture

All human breast cancer cell lines (MDA-MB-231 and BT-549) used in this study were maintained in DMEM (1×) (Dulbecco’s Modified Eagle Medium, Corning, USA) with 2 mM GlutaMAX™-I, 10% fetal bovine serum (FBS, Gibco, USA), and 100 units/mL penicillin/streptomycin. Cells were cultured in a humidified incubator at 37°C in the presence of 5% CO2.

### Cell line authentication using short tandem repeat (STR) analysis

Genomic DNA (gDNA) extracted from MDA-MB-231 and BT-549 TNBC cell lines was used for STR profiling. DNA amplification, including positive and negative controls, was performed with the AmpFLSTR Identifiler PCR amplification kit (Thermo Fisher Scientific, Waltham, MA, USA). The PCR products were then prepared for electrophoresis by adding Hi-Di Formamide and a size standard mixture to each sample and the allelic ladder. Electrophoresis was carried out using the Genetic Analyzer 3500xl DX system, and allelic calls were analyzed using GeneMapper software (Applied Biosystems/Thermo Fisher Scientific). Reference STR profiles were sourced from the American Type Culture Collection (ATCC) website (https://www.atcc.org/).

### Functional validation in TNBC models

To explore the functional role of selected circRNAs in regulating breast cancer biology, TNBC cells (MDA-MB-231 and BT-549) were transfected with selected siRNAs targeting circ_0001522, circ_0001278, and circ_0001801 junctions, compared to siControl (siCTRL), all purchased from Dharmacon. Sequences of siRNA used in current study are listed in Table S2. Transfection was performed using a reverse transfection approach as we previously described [5], at final siRNA concentration of 30 nM. After a 24-h incubation, complete DMEM was added to each well. To validate the knockdown efficiency of the selected circRNAs, total RNA was isolated from MDA-MB-231 and BT-549 cells at 72 h post transfection using the miRNeasy Mini kit (cat. no: 217004, Qiagen, Germany). RNA concentration and purity were measured using the NanoDrop 2000 (Thermo Scientific, DE, USA). Subsequently, 2 micrograms of total RNA were reverse transcribed into cDNA using the High-Capacity cDNA Reverse Transcription Kit and random hexamers (Applied Biosystems, Foster City, CA, USA), followed by RT-qPCR as described above.

### Colony-formation-unit assay (CFU)

The proliferative capacity of MDA-MB-231 and BT-549 cells was assessed using CFU assay and crystal violet staining in 5% SDS (Sigma Aldrich, St. Louis, MO, USA) under various treatment conditions. Cells were incubated in a 12-well plate for 5 days after siRNA-mediated knockdown of circ_0001522, circ_0001278, and circ_0001801. Wells were washed twice with PBS, stained, and then placed on a shaker for 30–60 min. After washing twice with tap water and air drying, images were captured and compared with siCTRL-treated cells. For quantification, each well was covered with 10% SDS solution followed by shaking for 30–60 min. Subsequently, 100 µL of the solution was transferred to a 96-well plate (in 6 replicates) and absorbance was measured at 590 nm using plate reader (NanoQuant, Infinite M200 Pro, Tecan).

### Dead/live cell staining using AO/EtBr fluorescent microscopy

Cell death was assessed using fluorescence microscopy and the AO/EtBr fluorescent staining method, which was applied to the transfected TNBC cells on day 5 post transfection. Cells were washed twice with PBS and stained for 2 min using a dual fluorescent solution containing 100 μg/mL AO and 100 μg/mL EtBr (AO/EtBr, Sigma Aldrich, St. Louis, MO, USA). The stained cells were then imaged using an Olympus IX73 fluorescence microscope (Olympus, Tokyo, Japan). Apoptotic cells were identified using AO staining, while EtBr-positive cells indicated the presence of necrotic cells.

### Three-dimensional and spheroid culture

3D organoid cultures were conducted following our previously described protocol [32]. In brief, 30,000 transfected MDA-MB-231 and BT-549 cells were mixed with 110 µL of Matrigel (Corning cat. no. 356231; Growth Factor Reduced (GFR) Basement Membrane Matrix). The cell suspension was then seeded as several drops onto pre-warmed (37 °C) 60 mm Ultra-Low Attachment Culture Dishes (Corning; 3261). The dishes were incubated upside-down at 37 °C and 5% CO_2_ for 20 min until the droplets solidified, after which 1 mL of expansion medium was added. Organoid formation was visualized and imaged under the microscope on day seven. For spheroid formation, transfected MDA-MB-231 and BT-549 cells were trypsinized, re-suspended in culture media, and were cultured in 60 mm low cell-binding dishes (Nunc, Thermo Scientific, Rockford, IL, USA) at a density of 3 × 10^4^ cells/well, as previously described [33]. After 10 days, multicellular tumor spheroids were imaged at ×10 magnification using an inverted microscope (Axio Observer-A1, Carl Zeiss, Germany).

### Wound healing and cell migration assay

To evaluate the migratory capacity of TNBC cells post-circRNA depletion, transfected cells were trypsinized on day 3 and reseeded in 6 cm culture dishes. Upon reaching confluence, a wound was created in the cell monolayer using a 200 μL plastic micropipette tip. The cells were washed, and the medium was replaced with fresh culture medium. Phase-contrast microscopy images of the wounded area were captured at 0 and 24 h. Wound area quantification was performed using ImageJ software (https://www.tessonics.com/products/imagel/). For Boyden chamber assay, cell migration was evaluated using an 8 µm pore-size BD transwell migration system as previously described [34]. Inserts were placed in a 12-well notched plate, and on day 3 post transfection, 4 × 10^4^ transfected cells in 500 μL of 1% FBS-DMEM were added to the upper chamber. The lower chamber contained 1.5 mL of either 1% FBS-DMEM (control) or 10% FBS-DMEM (chemoattractant). After 24 h, non-migrated cells on the upper surface of the inserts were removed with a cotton swab. The migrated cells were then fixed and stained using crystal violet staining in 5% SDS (Sigma Aldrich, St. Louis, MO, USA). Stained inserts were cut and mounted on microscope slides for analysis.

### Statistical analysis

Differential expression of circRNAs was analyzed using iDEP 2.0 with a fold-change cutoff of 2.0 and FDR < 0.05. The ten-year RFS was conducted using the ‘survival’ package in RStudio and Kaplan-Meier curves with log-rank tests in SPSS. Significance was set at *p* < 0.05, adjusting for age, tumor grade, and subtype. Graphing and statistical analyses were performed using GraphPad Prism v9. A two-tailed t-test was used for group comparisons (*p* < 0.05). Results are presented as mean ± SEM from triplicate experiments unless stated otherwise. RT-qPCR data were analyzed using the 2^−^^∆∆Ct^ method, normalizing to ACTB. Colony formation, migration, and invasion assay results were compared using t-tests or ANOVA. Statistical significance is marked as *p < 0.05, **p < 0.01, and ***p < 0.001.

## Supplementary information

Original Data

Table S1

Table S2

Table S3

Figure S1

Figure S2

Figure S3

Figure S4

## Data Availability

Processed data are provided in supplementary tables. The RNA transcriptomic data are available in the SRA repository under BioProject number PRJNA954402.
