# Supplementary material for: Circular RNA profiling identifies circ_0001522, circ_0001278, and circ_0001801 as predictors of unfavorable prognosis and drivers of triple-negative breast cancer hallmarks
Source: Cell Death Discov. 2025 Jul 9;11:316. doi: 10.1038/s41420-025-02576-9 (PMC12241340; doi:10.1038/s41420-025-02576-9)
Supplement: Supplementary file 1 — Original Data [file 41420_2025_2576_MOESM1_ESM.pdf]

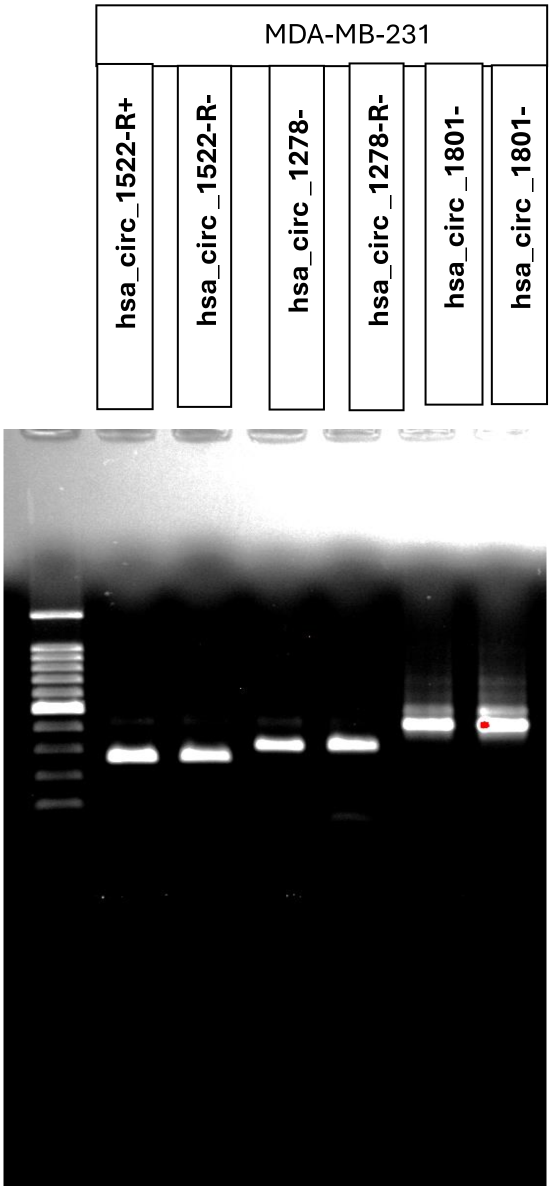
MDA-MB-231
hsa_circ_1522-R+
hsa_circ_1522-R-
hsa_circ_1278-
hsa_circ_1278-R-
hsa_circ_1801-
hsa_circ_1801-

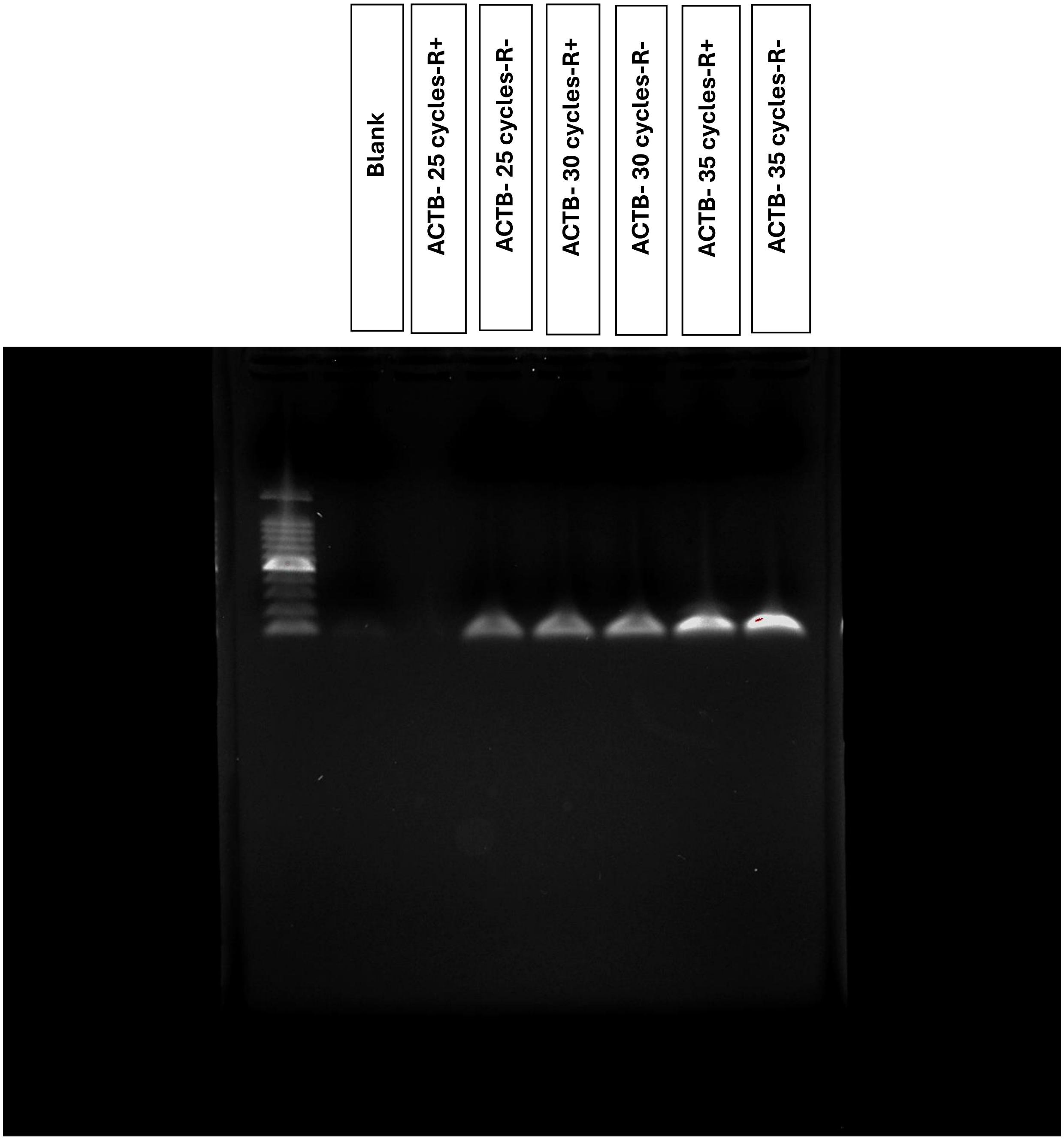
Blank
ACTB- 25 cycles-R+
ACTB- 25 cycles-R-
ACTB- 30 cycles-R+
ACTB- 30 cycles-R-
ACTB- 35 cycles-R+
ACTB- 35 cycles-R-
