# Supplementary material for: Circular RNA profiling identifies circ_0001522, circ_0001278, and circ_0001801 as predictors of unfavorable prognosis and drivers of triple-negative breast cancer hallmarks
Source: Cell Death Discov. 2025 Jul 9;11:316. doi: 10.1038/s41420-025-02576-9 (PMC12241340; doi:10.1038/s41420-025-02576-9)
Supplement: Supplementary file 2 — Table S1 [file 41420_2025_2576_MOESM2_ESM.docx]

| Primers ID | Sequence | Size |
| --- | --- | --- |
| hsa_circ_0001522_F | AACACTCGAGGAACTGGGTC | 427 bp |
| hsa_circ_0001522_R | AAGACAAGCACACTGAAACAGG |  |
| hsa_circ_0001278_F | AGAACTTTGGAACCAAGGAGCA | 270 bp |
| hsa_circ_0001278_R | TCATCAACCCTGGGTAAACAGT |  |
| hsa_circ_0001801_F | TCCACTTGTCAGCACCTTGC | 324 bp |
| hsa_circ_0001801_R | CTCTGAAGGCTTGCTCCACT |  |

Table S1. Primers for SYBRGreen RT-qPCR assays
