# Supplementary material for: Circular RNA profiling identifies circ_0001522, circ_0001278, and circ_0001801 as predictors of unfavorable prognosis and drivers of triple-negative breast cancer hallmarks
Source: Cell Death Discov. 2025 Jul 9;11:316. doi: 10.1038/s41420-025-02576-9 (PMC12241340; doi:10.1038/s41420-025-02576-9)
Supplement: Supplementary file 3 — Table S2 [file 41420_2025_2576_MOESM3_ESM.docx]

| Name | Target Sequence | Supplier |
| --- | --- | --- |
| siRNA-CTM-1004004  APOMB-000015  For hsa_circ_0001522 | CUGGAGCUCUCUAUCAAUAUU | Dharmacon |
| siRNA-CTM-1004008  APOMB-000021  For hsa_circ_0001278 | UGGGUUUAACUAUAGAUCAUU | Dharmacon |
| siRNA-CTM-1004009  APOMB-000023  For hsa_circ_000801 | UGGGCUUAAUUUUAGGAUUUU | Dharmacon |
| D-001810-01-05  ON-TARGET plus Non-targeting siRNA | UGGUUUACAUGUCGACUAA | Dharmacon |

Table S2. siRNA sequence used for circRNA knockdown
