# Supplementary material for: Circular RNA profiling identifies circ_0001522, circ_0001278, and circ_0001801 as predictors of unfavorable prognosis and drivers of triple-negative breast cancer hallmarks
Source: Cell Death Discov. 2025 Jul 9;11:316. doi: 10.1038/s41420-025-02576-9 (PMC12241340; doi:10.1038/s41420-025-02576-9)
Supplement: Supplementary file 5 — Figure S1 [file 41420_2025_2576_MOESM5_ESM.pdf]

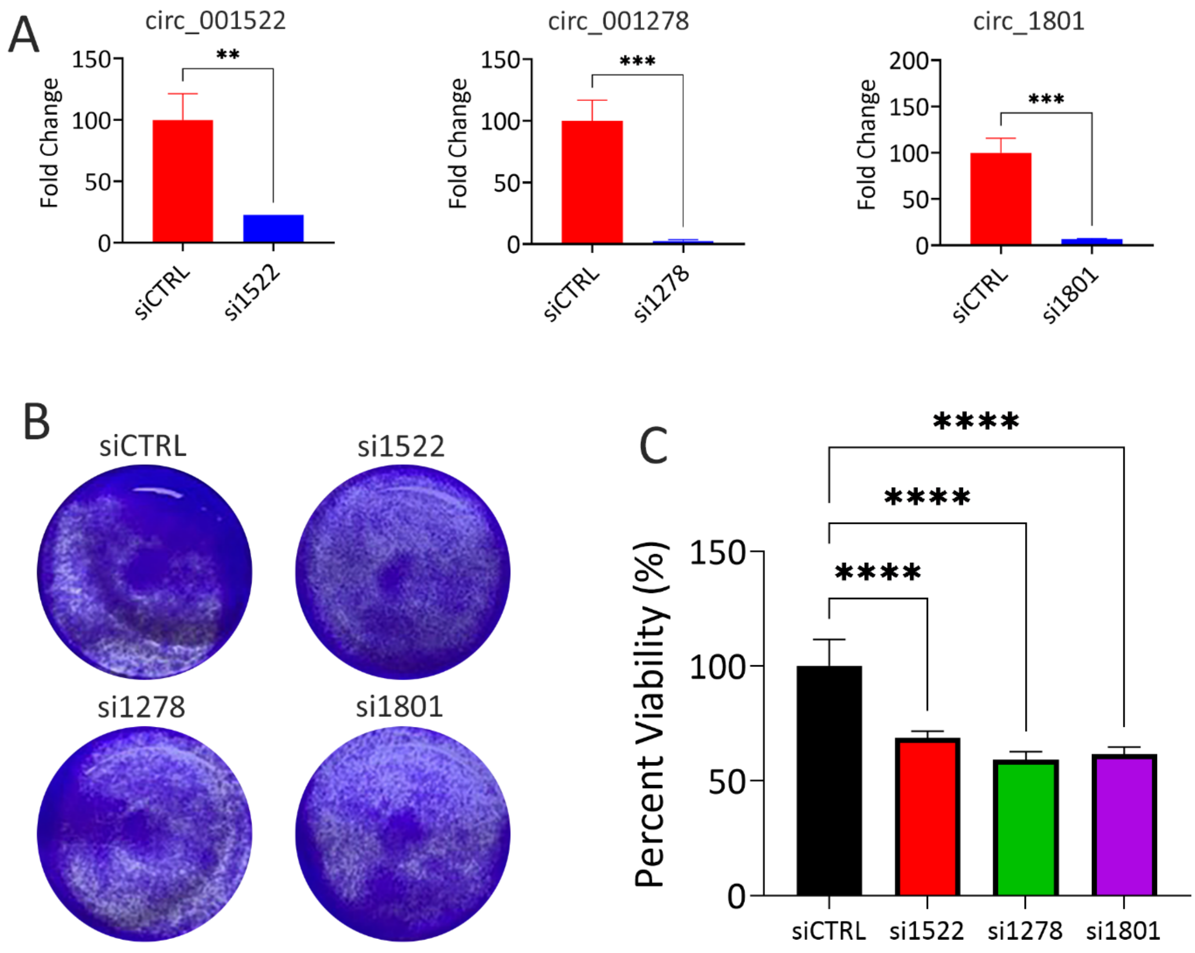

Figure S1. Suppression of circ_0001522, circ_0001278, and circ_0001801 impairs BT-549 colony forming unit (CFU) potential. (A) Efficient knockdown of circ_0001522, circ_0001278, and circ_0001801 using siRNAs targeting the respective backsplice junctions in BT-549. Data are shown as mean ± S.E.M., n = 6. **(B)** Representative images from colony-forming unit (CFU) assay demonstrating suppression of CFU potential in BT-549 cells by siRNA-mediated silencing of hsa_circ_0001522, hsa_circ_0001278, and hsa_circ_0001801. **(C)** Quantification of BT-549 CFU potential following depletion of the indicated circRNAs. Data are shown as mean ± S.E.M., n = 6 from three independent experiments.
