# Supplementary material for: Circular RNA profiling identifies circ_0001522, circ_0001278, and circ_0001801 as predictors of unfavorable prognosis and drivers of triple-negative breast cancer hallmarks
Source: Cell Death Discov. 2025 Jul 9;11:316. doi: 10.1038/s41420-025-02576-9 (PMC12241340; doi:10.1038/s41420-025-02576-9)
Supplement: Supplementary file 6 — Figure S2 [file 41420_2025_2576_MOESM6_ESM.pdf]

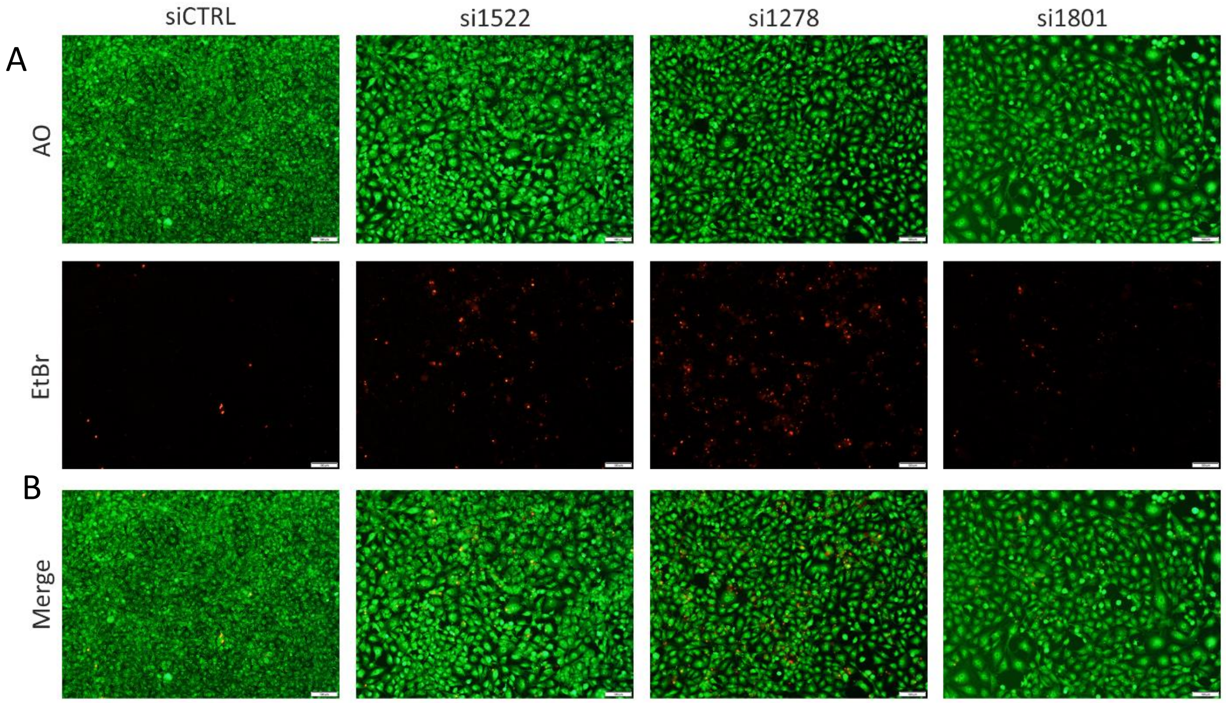

Figure S2. Live and dead staining of circRNA knockdown in BT-549 TNBC cells. Representative images showing AO (upper panel), EtBr (middle panel), and merged (lower panel) staining in BT-549 cells transfected with siCirc_0001522, siCirc_0001278, and siCirc_0001801 on day 6 post-transfection.
