# Supplementary material for: Circular RNA profiling identifies circ_0001522, circ_0001278, and circ_0001801 as predictors of unfavorable prognosis and drivers of triple-negative breast cancer hallmarks
Source: Cell Death Discov. 2025 Jul 9;11:316. doi: 10.1038/s41420-025-02576-9 (PMC12241340; doi:10.1038/s41420-025-02576-9)
Supplement: Supplementary file 7 — Figure S3 [file 41420_2025_2576_MOESM7_ESM.pdf]

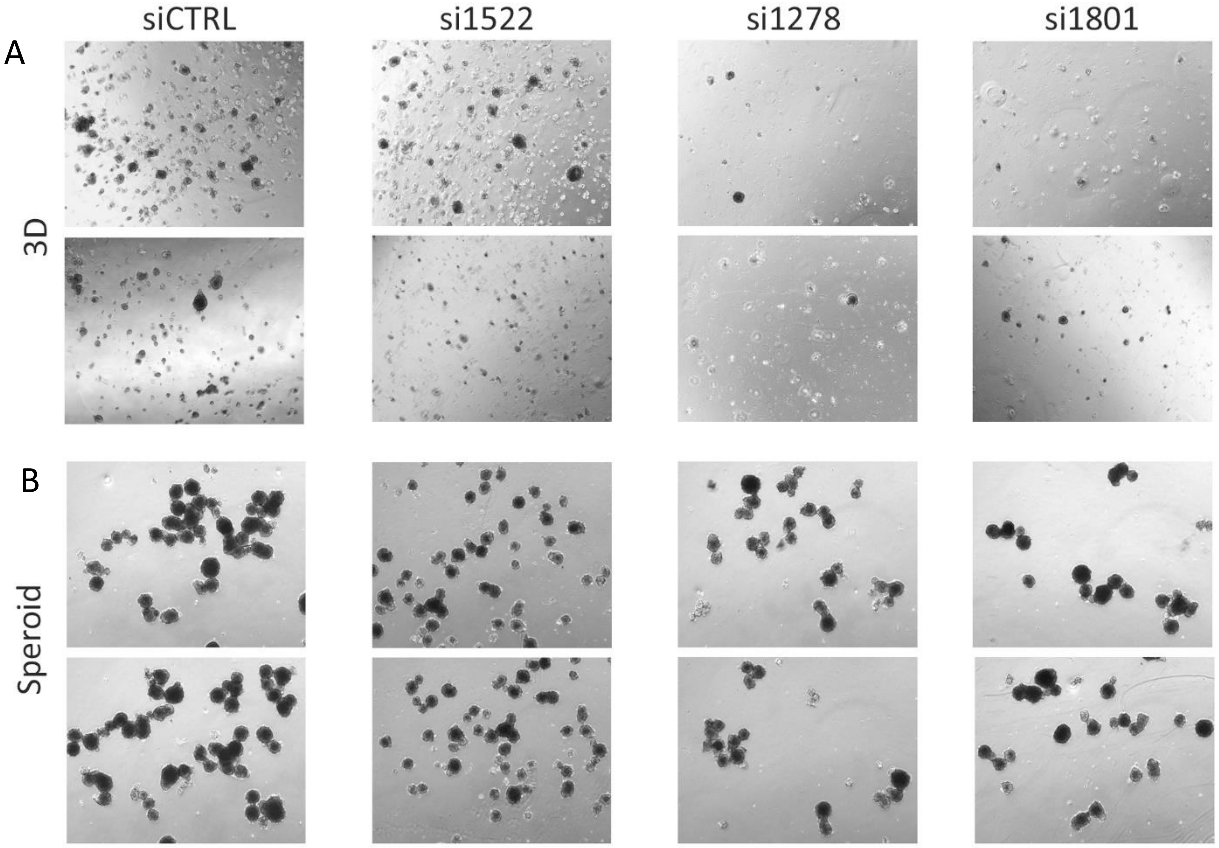

Figure S3. Targeted depletion of circ_0001522, circ_0001278, and circ_0001801 inhibits three-dimensional and spheroid growth of BT-549 TNBC. (A) Representative images showing suppression of BT-549 cell growth in three-dimensional (3D) Matrigel cultures following depletion of the indicated circRNAs. **(B)** Representative images showing suppression of spheroid formation in BT-549 cells after depletion of circ_0001522, circ_0001278, and circ_0001801 compared to siCTRL cells.
