# Supplementary material for: Circular RNA profiling identifies circ_0001522, circ_0001278, and circ_0001801 as predictors of unfavorable prognosis and drivers of triple-negative breast cancer hallmarks
Source: Cell Death Discov. 2025 Jul 9;11:316. doi: 10.1038/s41420-025-02576-9 (PMC12241340; doi:10.1038/s41420-025-02576-9)
Supplement: Supplementary file 8 — Figure S4 [file 41420_2025_2576_MOESM8_ESM.pdf]

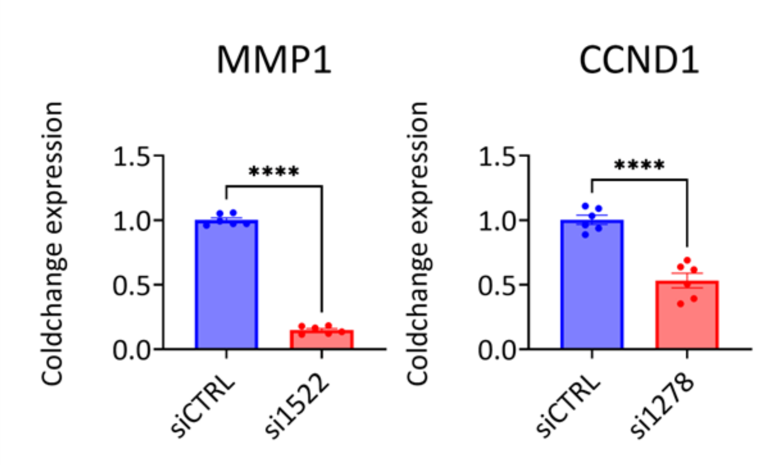

Figure S4. Validation of selected gene targets in circRNA knockdown cells. RT-qPCR analysis of MMP1 (left panel) and CCND1 (right panel) expression in MDA-MB-231 cells following the knockdown of circ_001522 and circ_001278, respectively. Data are presented as mean ± S.E.M., n = 6. Statistical significance was assessed using a t-test. **** $p < 0.00005$.
